# Mental health service utilization in publicly insured survivors of childhood cancer: a claims-based analysis

**DOI:** 10.1093/jncics/pkaf099

**Published:** 2025-10-14

**Authors:** Xu Ji, Xin Hu, Ilana Graetz, Karen E Effinger, Jordan Gilleland Marchak, Janet R Cummings

**Affiliations:** Department of Pediatrics, Emory University School of Medicine, Atlanta, GA, United States; Aflac Cancer and Blood Disorders Center, Children’s Healthcare of Atlanta, Atlanta, GA, United States; Department of Health Policy and Management, Emory University Rollins School of Public Health, Atlanta, GA, United States; Department of Health Policy and Management, Emory University Rollins School of Public Health, Atlanta, GA, United States; Department of Radiation Oncology, Emory University School of Medicine, Atlanta, GA, United States; Department of Health Policy and Management, Emory University Rollins School of Public Health, Atlanta, GA, United States; Department of Radiation Oncology, Emory University School of Medicine, Atlanta, GA, United States; Department of Pediatrics, Emory University School of Medicine, Atlanta, GA, United States; Aflac Cancer and Blood Disorders Center, Children’s Healthcare of Atlanta, Atlanta, GA, United States; Department of Pediatrics, Emory University School of Medicine, Atlanta, GA, United States; Aflac Cancer and Blood Disorders Center, Children’s Healthcare of Atlanta, Atlanta, GA, United States; Department of Health Policy and Management, Emory University Rollins School of Public Health, Atlanta, GA, United States

## Abstract

**Background:**

Childhood cancer survivors face long-term psychological challenges, including depression, trauma/stress, and anxiety. However, objective assessments of mental health service utilization among child and young adult (YA) survivors of childhood cancer remain limited. We examined mental health care utilization among publicly insured childhood cancer survivors and disparities by sociodemographic and neighborhood-level factors.

**Methods:**

Using multistate public insurance claims data, we identified 5946 survivors (diagnosed ≤21 years) who completed cancer therapy; initiated treatment episode(s) for depression, trauma/stress, or anxiety post cancer therapy; and maintained continuous coverage. Logistic regressions examined factors associated with having any mental health visit and ≥4 visits within 12 weeks of treatment episode initiation in children (ages 3-17) and YAs (ages 18-39).

**Results:**

Among 4052 child treatment episodes, 54.6% were in female survivors, 41.5% non-Hispanic White survivors, and 27.4% Hispanic survivors; demographics were similar across 3871 YA episodes. Utilization was highest among survivors aged 3–11 years (any visit: 73.4%; ≥4 visits: 39.8%), followed by those aged 12–17 years (67.8%; 33.2%), 18–26 years (51.9%; 20.2%), and 27–39 years (43.3%; 16.4%). Hispanic children were less likely than non-Hispanic White peers to have ≥4 mental health visits (marginal effect = –8.73 percentage points; 95% CI = –12.78 to –4.68), as were children in most (vs least) deprived neighborhoods (marginal effect = –8.80 percentage points; 95% CI = –14.07 to –3.53). Similar disparities were observed for any mental health visit.

**Conclusion:**

Mental health service utilization was low among publicly insured childhood cancer survivors after mental health diagnosis, with notable disparities by age, ethnicity, and geographic location, underscoring the need for interventions to improve psychological support in this underserved population.

## Introduction

With medical advances, the number of childhood cancer survivors is rising, approaching 580 000 in the United States.^1^ These survivors often face long-term mental health challenges, including depression, trauma/stress, and anxiety.^2-4^ Such challenges can arise from the toxicity of cancer treatment, including physical (eg, infertility, chronic medical conditions) and psychological effects (eg, fear of cancer recurrence and second cancers).^5-7^ For young adults (YAs), a childhood cancer diagnosis further complicates their transition to adulthood by interrupting education, career development, and relationship formation, increasing the risk of adverse mental health outcomes.^2^^,^^8^

Mental health services refer to a range of psychological, behavioral, and social support interventions—such as psychotherapy, counseling, and case management—intended to help survivors manage conditions such as anxiety and depression that arise during and after the cancer experience.^9^ They are recommended as guideline-concordant care for survivors of childhood cancer, including YAs, who often experience a broad spectrum of challenges, including mental health concerns, educational and vocational difficulties, and fatigue.^4^^,^^8^^,^^10^^,^^11^ Evaluating utilization of mental health services is therefore essential to ensure childhood cancer survivors receive appropriate, guideline-based care.

Medicaid is the largest single-payer of mental health services in the United States.^12^ Medicaid and Children’s Health Insurance Program (CHIP) are the largest insurer of children, covering >35 million children in 2023.^13^ Yet, Medicaid/CHIP enrollees often face barriers to mental health services, including a shortage of specialists who accept Medicaid, prolonged wait times for appointments, inadequate transportation, and caregivers’ limited availability.^14-16^ Even after initiating care, maintaining mental health services remains challenging, with high rates of treatment discontinuation commonly observed among Medicaid-enrolled children.^17-19^ Yet, little is known about the use of mental health services among publicly insured childhood cancer survivors. Understanding utilization patterns and disparities for this economically disadvantaged, high-need population is critical to informing survivorship care planning, resource allocation, and policy reforms to optimize survivorship outcomes.

Furthermore, nationwide or multistate data on mental health service utilization among childhood cancer survivors are limited, hindering efforts to evaluate uptake of guideline-concordant mental health care, identify disparities across geography and population characteristics, and develop interventions to address care gaps. Existing national estimates largely rely on self-reported survey data,^20-22^ which are subject to recall and nonresponse biases, and focus primarily on survivors of adult-onset cancers, whose psychological response to cancer differs from those of childhood cancer survivors.^23^ Research on mental health service use specifically among survivors of childhood cancer is limited; the few studies on this population are survey-based and/or restricted to selected cohorts of adult survivors.^24^^,^^25^ To date, multistate claims-based assessments of mental health service utilization among young survivors of childhood cancer remain lacking.

To fill the evidence gap, we analyzed multistate claims data to examine mental health service visits among publicly insured childhood cancer survivors aged 3-39 years with a mental health diagnosis. We assessed visits separately for children (3-17 years) and YAs (18-39 years), considering the distinct psychological effects of cancer across developmental stages. We also identified disparities in service visits by sociodemographic and county-level characteristics.

## Methods

### Data and study cohort

We used the 2000-2015 Medicaid Analytic eXtract (MAX) files and 2016-2020 Transformed Medicaid Statistical Information System Analytic Files (TAF) administered by the Centers for Medicare and Medicaid Services (CMS).^26^^,^^27^ These files provide person-level data on Medicaid and CHIP enrollment, demographics, eligibility type, and detailed health care visit records across outpatient, inpatient, and pharmacy settings.^26^^,^^27^ We also merged county-level measures from the Area Health Resources Files^28^ and the Social Deprivation Index County Files.^29^ This study was approved by the Institutional Review Board of Emory University.

Our cohort was derived in several steps. First, we identified childhood cancer survivors using a claims-based algorithm suggested in prior studies,^30-34^ defined as individuals who: (1) had a diagnosis of hematological (leukemia, lymphoma), central nervous system (CNS), bone or soft tissue, or gonadal cancer—the most prevalent childhood cancer types in the United States;^35^ (2) were aged ≤21 years at first claim with a cancer diagnosis; and (3) completed cancer therapy between 2000-2020. Cancer diagnosis required ≥2 outpatient and/or inpatient claims on distinct dates, with International Classification of Diseases (ICD) codes falling within relevant Clinical Classifications Software categories (Table S1).^36^ Cancer therapy (surgery, chemotherapy, radiation therapy, hematopoietic stem cell transplantation) was identified using the Current Procedural Terminology/Healthcare Common Procedure Coding System procedure codes or National Drug Codes. Completion of cancer therapy was defined as 30 days after the last inpatient or outpatient claim for cancer therapy or conclusion of the final oral chemotherapy prescription, whichever occurred later.^30^

**Figure 1.**
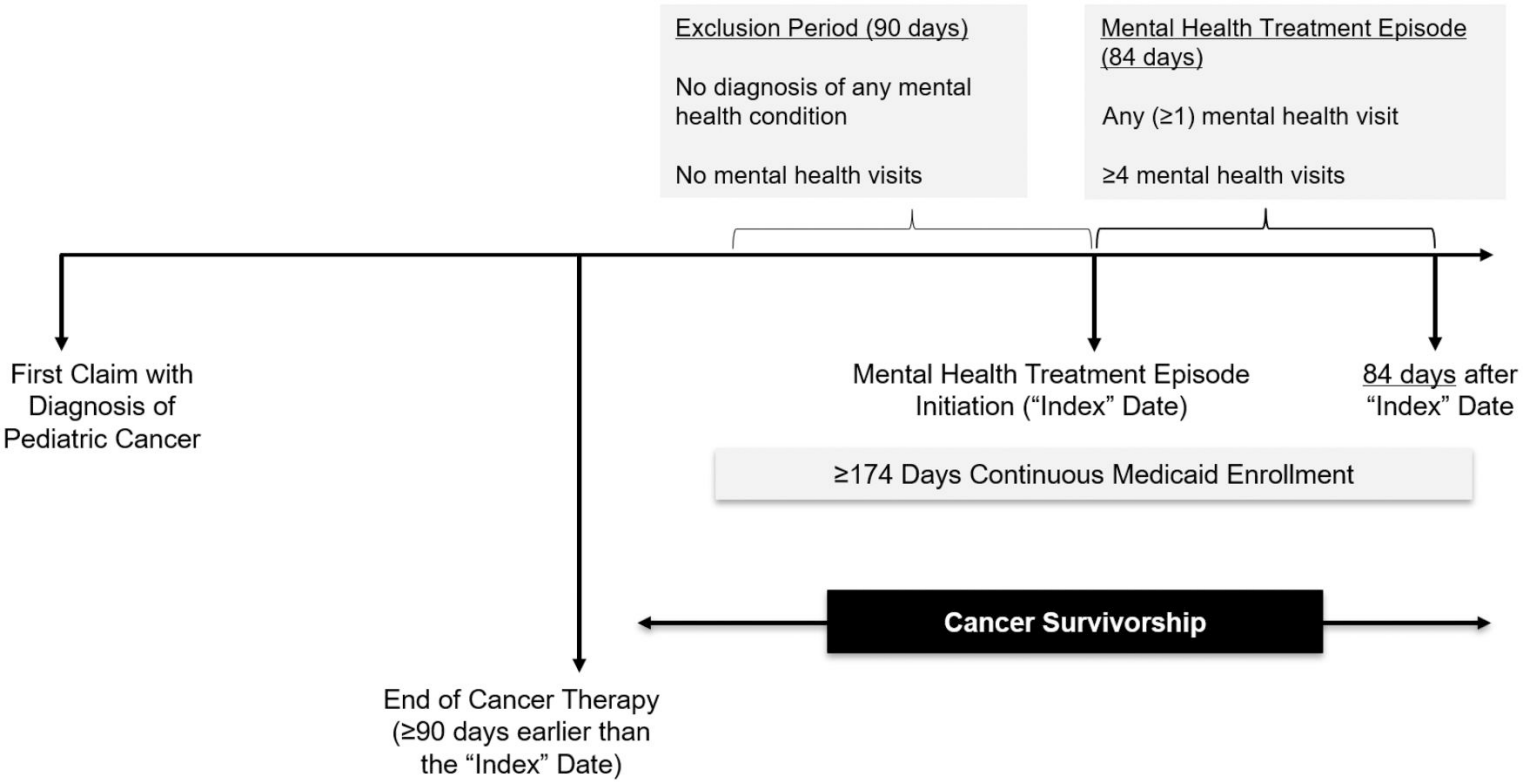
Mental health treatment episode identification.

Second, we restricted our cohort to those with an incident diagnosis for depression, anxiety, or trauma/stress between 2016 and 2020. These conditions are highly prevalent in childhood cancer survivors, for whom mental health services are guideline-recommended.^4^^,^^10^ Consistent with prior claims-based literature,^17^^,^^37-39^ diagnosis of a mental health condition required ≥1 inpatient claim or ≥2 outpatient claims on distinct dates within 84 days of the first claim, with corresponding ICD diagnosis codes (Table S2). The first of these claims marked the “index date.” A mental health treatment episode (“treatment episode” hereafter) was defined as the 84 days after the index date (Figure 1). To ensure incident mental health diagnoses, we required a 90-day “exclusion period” before the index date, during which there was no health care encounter related to any mental health diagnosis (Table S2) or any mental health-related procedure (Table S3).^17^^,^^18^^,^^38^ Because our focus was on mental health service use during cancer survivorship, we required the index date to be ≥90 days after completion of cancer therapy to ensure that all exclusion periods and treatment episodes occurred post cancer therapy.

Third, we further restricted to survivors aged 3-39 years throughout each treatment episode, consistent with the National Cancer Institute’s definition of children and YAs.^40^ Notably, survivors could have multiple treatment episodes, in which case the end date of one episode (84 days from the index date) had to be ≥90 days before the index date of the subsequent episode. For each treatment episode, continuous Medicaid or CHIP enrollment was required from 90 days before through 84 days after the index date, allowing administrative gaps of <30 days (Figure 1).^41-43^ Moreover, we excluded enrollees missing county-level data or covered by Medicare. Lastly, we restricted our analysis to 43 states and the District of Columbia, where TAF Other Services Files did not have “high concern” quality issues.^44^ The sample derivation process is detailed in Figure S1.

### Outcomes

Mental health service utilization was measured by a dichotomous indicator for survivors who had any (≥1) outpatient health care claim with a mental health-related procedure code (including psychotherapy, other psychosocial interventions, case management, or other mental health services; Table S3) within 84 days of the index date (“any mental health visit” hereafter).^17^^,^^38^^,^^45^ We also created a dichotomous measure for receiving ≥4 mental health visits on distinct days within the 84-day period.^17^^,^^18^^,^^38^ This 4-visit threshold aligns with prior studies,^17^^,^^37^^,^^38^ drawing on evidence that brief interventions (ie, within 4 sessions) can yield improvements in symptoms of depression and/or anxiety among youth.^46^^,^^47^ Notably, the 4-visit threshold represents a conservative (lower-bound) number of visits that should be received to allow for symptom improvement, but it does not necessarily capture a full course of mental health treatment.^47^

### Covariates

Covariates were guided by Andersen’s Behavioral Model of Health Services Use.^48-50^ Individual-level covariates included predisposing characteristics (age group at index date [3-11, 12-17, 18-26, 27-39 years],^51^^,^^52^ sex, race/ethnicity), enabling factors (Medicaid eligibility type and plan type, both measured in the month of index date), and need-related factors (mental health conditions during treatment episodes, cancer type, and years since cancer therapy completion^53^). Area-level covariates included rurality of residence, designation as mental health professional shortage areas, and Social Deprivation Index (SDI). Social Deprivation Index was categorized into quartiles based on the distribution of all counties across the United States, with Quartiles 1 (Q1) and 4 (Q4) representing the least deprived and most deprived areas, respectively.

### Statistical analyses

Our analyses were conducted at the survivor-episode level. We first described sample characteristics and unadjusted outcome percentages by sociodemographic and clinical factors. As our outcomes were binary, multiple logistic regressions were used to identify the factors associated with each outcome,^54^ adjusting for individual- and county-level covariates, year of treatment episode initiation, and state. Standard errors were clustered at the individual level to account for correlation across multiple treatment episodes from the same survivor.^55^ Our analyses were conducted separately for children and YAs.

Marginal effects (MEs) and 95% confidence intervals (CIs) were calculated using the “margins” macro command in SAS statistical software. Marginal effect was interpreted as the model-adjusted difference in the percentage of an outcome (eg, any mental health visit) between the group of interest (eg, Hispanic survivors) and the reference group (eg, non-Hispanic White survivors) for a given covariate (eg, race/ethnicity), holding all other covariates at their observed values.^56^ For ease of interpretation, we also calculated the relative change by dividing the ME by the model-adjusted visit probability in the reference group. Analyses used SAS Enterprise Guide 7.1 available through the Virtual Research Data Center of the CMS. Statistical significance was determined with 2-sided tests at 0.05. We followed the Strengthening the Reporting of Observational Studies in Epidemiology (STROBE) reporting guideline.

## Results

### Sample characteristics

We identified 5946 survivors, with a total of 7923 treatment episodes. Of the 4052 treatment episodes identified in children, more than half were from female survivors (54.6%), enrolled due to low income (67.6%), and residents of metropolitan areas (82.0%; Table 1). Approximately two-fifths were non-Hispanic White survivors (41.5%), whereas 27.4% were Hispanic survivors and 8.4% were non-Hispanic Black survivors. Hematological cancer was most prevalent (57.1%), followed by CNS tumor (25.4%) and bone or soft tissue cancer (14.4%). More than half (52.8%) of episodes occurred 2 to 5 years post cancer therapy, with 30.3% more than 5 years post-therapy and 16.9% less than 2 years post-therapy.

**Table 1.** Sample characteristics of mental health treatment episodes.

Characteristics	Child survivors ages 3-17 years		Young adult survivors ages 18-39 years	
	*n*	%	*n*	%
Total observations	4052		3871	
Age group at index date				
3-11 years	1352	33.4	.	.
12-17 years	2700	66.6	.	.
18-26 years	.	.	2830	73.1
27-39 years	.	.	1041	26.9
Sex				
Male	1838	45.4	1448	37.4
Female	2214	54.6	2423	62.6
Race/Ethnicity				
Hispanic	1112	27.4	831	21.5
Non-Hispanic Black	342	8.4	471	12.2
Non-Hispanic other, multiple, or unknown race/ethnicity^a^	918	22.7	751	19.4
Non-Hispanic White	1680	41.5	1818	47.0
Medicaid eligibility type^b^				
Low income	2738	67.6	2543	65.7
Disability	1226	30.3	1235	31.9
Other or unknown eligibility type	88	2.2	93	2.4
Medicaid plan type^c^				
Comprehensive managed care organization	3116	76.9	2994	77.3
Behavioral health organization, prepaid health plan, or other plan types	522	12.9	475	12.3
Primary care case management or fee-for-service	414	10.2	402	10.4
Presence of mental health conditions during episode				
Depression only	917	22.6	1135	29.3
Stress/trauma only	1399	34.5	357	9.2
Anxiety only	851	21.0	1046	27.0
Two diagnoses	790	19.5	1194	30.8
Three or more diagnoses	95	2.3	139	3.6
Episode initiation year				
2016	640	15.8	603	15.6
2017	704	17.4	641	16.6
2018	918	22.7	798	20.6
2019	1008	24.9	879	22.7
2020	782	19.3	950	24.5
Cancer type (first cancer diagnosis)				
Hematological	2314	57.1	2040	52.7
Bone or connective tissue	585	14.4	544	14.1
Central nervous system	1030	25.4	871	22.5
Gonadal	123	3.0	416	10.7
Time from end-of-cancer-therapy to episode initiation^d^				
90 days to <2 years	686	16.9	344	8.9
2-5 years	2140	52.8	1409	36.4
>5 years	1226	30.3	2118	54.7
County-level SDI^e^, quartiles				
Q1: least deprived areas	534	13.2	496	12.8
Q2	860	21.2	792	20.5
Q3	1088	26.9	1108	28.6
Q4: most deprived areas	1570	38.7	1475	38.1
County-level metro status^f^				
Non-metro (including rural) areas	731	18.0	779	20.1
Metropolitan areas	3321	82.0	3092	79.9
County-level mental health professional shortage areas^g^				
None	197	4.9	193	5.0
Partial	2825	69.7	2637	68.1
Full	1030	25.4	1041	26.9

Abbreviations: SDI = Social Deprivation Index; ME = marginal effects.

a This group includes non-Hispanic American Indian or Alaskan Native, non-Hispanic Asian or Pacific Islander, non-Hispanic multiracial group, or unknown race/ethnicity.

b Eligibility information extracted in the month of episode initiation.

c Plan type information extracted in the month of episode initiation.

d We defined survivors <2 years post-cancer therapy as early survivors and those >5 years post-cancer therapy as long-term survivors.

e SDI generated based on American Community Survey data. Year-specific SDIs were available from 2016 to 2019. Year-specific SDI was linked for individuals identified in 2016-2019. SDI from 2019 was used to link with individuals identified in 2020. Data available from: https://www.graham-center.org/maps-data-tools/social-deprivation-index.html.

f Defined based on 2013 Rural-Urban Continuum Codes. Metro includes codes 1, 2, 3; Non-metro Urban includes codes 4, 5, 6, 7; Rural includes code 8, 9. For detailed documentation for each code, see: https://www.ers.usda.gov/data-products/rural-urban-continuum-codes/documentation/.

g Information extracted from 2020 and 2021 Area Health Resources Files. Year-specific county-level measures were linked for individuals identified in 2016-2020 (data available from: https://data.hrsa.gov/topics/health-workforce/nchwa/ahrf). An area with a mental health provider shortage is designated by the US Health Resources and Services Administration (HRSA). This designation is determined using a federal scoring system that evaluates multiple factors, including the ratio of mental health providers to the population, poverty levels, age distribution, prevalence of substance use, and travel time or distance to the nearest source of care. Mental health professional shortage areas may cover entire geographic regions, specific population groups (eg, low-income residents), or particular facilities. Reference: https://bhw.hrsa.gov/workforce-shortage-areas/shortage-designation/scoring.

Of the 3871 episodes identified in YAs, the sociodemographic and cancer type distributions were similar to those among children (Table 1). For YAs, more than half (54.7%) of their episodes were more than 5 years post-therapy, with 36.4% within 2 to 5 years post-therapy and 8.9% less than 2 years post-therapy.

### Receipt of any mental health visit

The likelihood of receiving any mental health visit during treatment episode was highest among survivors aged 3-11 years (73.4%), followed by those aged 12-17 years (67.8%), 18-26 years (51.9%), and 27-39 years (43.3%; Tables 2-3, Figure S2). This pattern persisted in adjusted models including both children and YAs (Table S4): compared with the 3-11 year age group (model-adjusted percentage = 71.09%), those aged 12-17, 18-26, and 27-39 had a 4.32 percentage point (ppt, 95% CI = –7.60 to –1.03, *P *= .010), 17.23 ppt (95% CI = –20.73 to –13.73, *P *< .001), and 25.93 ppt (95% CI = –30.67 to –21.19, *P *< .001) lower likelihood of any visit, respectively. These absolute differences correspond to relative reductions of 6.1%, 24.2%, and 36.5% (calculated as each ME divided by the model-adjusted percentage of any visit in the 3-11 year reference group).

**Table 2.** Factors associated with utilization of mental health services among child survivors ages 3-17 years.

Characteristics	Any mental health visit			≥4 Mental health visits		
	Unadjusted percentage (%)^1^	Adjusted differences		Unadjusted percentage (%)^1^	Adjusted differences	
		ME^2^	95% CI		ME^2^	95% CI
Age groups at index date						
3-11 years	73.4	Ref		39.8	Ref	
12-17 years	67.8	−4.15*	(–7.42, –0.88)	33.2	−4.53*	(–8.00, –1.06)
Sex						
Male	69.1	Ref		35.6	Ref	
Female	70.1	0.96	(–2.01, 3.92)	35.3	−0.20	(–3.29, 2.88)
Race/Ethnicity						
Hispanic	60.8	−6.80***	(–10.76, –2.84)	27.1	−8.73***	(–12.78, –4.68)
Non-Hispanic Black	72.8	3.08	(–2.60, 8.77)	36.5	−0.40	(–6.49, 5.70)
Non-Hispanic other, multiple, or unknown race/ethnicity	73.5	1.76	(–2.10, 5.61)	37.5	−0.83	(–4.92, 3.26)
Non-Hispanic White	72.8	Ref		39.6	Ref	
Medicaid eligibility type						
Low-income	72.2	Ref		37.6	Ref	
Disability	63.4	−7.82***	(–11.15, –4.49)	30.3	−6.16***	(–9.47, –2.86)
Other or unknown eligibility type	79.5	9.51*	(1.99, 17.03)	38.6	4.36	(–6.13, 14.84)
Medicaid plan type						
Comprehensive managed care organization	69.5	Ref		35.9	Ref	
Behavioral health organization, prepaid health plan, or other	76.6	6.26**	(2.21, 10.31)	37.4	0.28	(–4.37, 4.94)
Primary care case management or fee-for-service	62.1	−11.63***	(–16.89, –6.38)	29.5	−8.66***	(–13.27, –4.06)
Presence of mental health conditions during episode						
Depression only	61.4	Ref		27.7	Ref	
Stress/trauma only	80.4	16.65***	(12.54, 20.77)	44.2	13.93***	(9.76, 18.10)
Anxiety only	55.0	−8.15**	(–13.05, –3.25)	26.9	−2.75	(–7.15, 1.65)
Two diagnoses	73.7	11.36***	(6.87, 15.85)	35.4	6.93**	(2.39, 11.47)
Three or more diagnoses	89.5	24.98***	(17.41, 32.55)	55.8	24.53***	(13.90, 35.16)
Episode initiation year						
2016	69.8	Ref		33.0	Ref	
2017	67.3	−1.94	(–6.67, 2.79)	32.0	1.94	(–2.80, 6.67)
2018	68.3	−0.32	(–-4.86, 4.23)	34.5	5.58*	(0.93, 10.24)
2019	72.5	2.97	(–1.46, 7.41)	38.8	3.54	(–1.33, 8.40)
2020	69.6	−0.63	(–5.38, 4.12)	37.2	2.52	(–2.03, 7.06)
Cancer type (first cancer diagnosis)						
Hematological	69.6	Ref		35.1	Ref	
Bone or connective tissue	69.1	−1.45	(–5.74, 2.84)	34.2	−1.07	(–5.46, 3.31)
Central nervous system	71.3	2.18	(–1.28, 5.64)	37.6	2.62	(–1.06, 6.31)
Gonadal	60.2	−7.94	(–16.85, 0.97)	29.3	−4.11	(–12.45, 4.22)
Time from end-of-cancer therapy to episode initiation						
90 days to <2 years	67.1	Ref		33.8	Ref	
2-5 years	70.3	3.72	(–0.18, 7.61)	36.3	2.77	(–1.24, 6.79)
>5 years	70.1	4.37	(–0.05, 8.78)	34.7	2.58	(–2.07, 7.23)
County-level SDI, Quartiles						
Q1: least deprived areas	78.1	Ref		44.6	Ref	
Q2	72.4	−2.84	(–8.22, 2.53)	39.7	−1.92	(–7.46, 3.62)
Q3	71.0	−3.15	(–8.19, 1.89)	35.8	−4.46	(–9.82, 0.89)
Q4: most deprived areas	64.4	−7.95**	(–13.04, –2.87)	29.7	−8.80**	(–14.07, –3.53)
County-level metro status						
Non-metro (including rural) areas	73.3	Ref		36.4	Ref	
Metropolitan areas	68.9	3.20	(–1.31, 7.70)	35.2	3.26	(–1.03, 7.56)
County-level mental health professional shortage areas						
None	81.7	Ref		46.7	Ref	
Partial	66.6	−10.59**	(–16.89, –4.28)	33.8	−6.89	(–14.12, 0.34)
Full	75.8	−0.91	(–7.54, 5.72)	37.6	−2.54	(–10.23, 5.15)

Abbreviations: ME = marginal effects; CI = confidence interval; Ref = reference; SDI = Social Deprivation Index.

*n* = 4052 episodes. All covariates listed in this table were included as control variables in the regression models. Regression models also adjusted for state indicators.

a Unadjusted percentage refers to the proportion of individuals within the subgroup specified in each row (eg, female survivors)—that is, using that subgroup as the denominator—who experienced the study outcome (eg, had any mental health visit).

b MEs were interpreted as the model-adjusted difference in the percentage of an outcome (eg, having any mental health visit) between the group of interest (eg, Hispanic survivors) and the reference group (eg, non-Hispanic White survivors) for a given covariate (eg, race/ethnicity), holding all other covariates at their observed values.

* *P *< .05.

** *P *< .01.

*** *P *< .001.

**Table 3.** Factors associated with utilization of mental health services among young adult survivors ages 18-39 years.

Characteristics	Any mental health visit			≥4 Mental health visits		
	Unadjusted percentage (%)^1^	Adjusted differences		Unadjusted percentage (%)^1^	Adjusted differences	
		ME^2^	95% CI		ME^2^	95% CI
Age groups at index date						
18-26 years	51.9	Ref		20.2	Ref	
27-39 years	43.3	−7.70***	(–11.79, –3.62)	16.4	−3.41*	(–6.45, –0.37)
Sex						
Male	51.0	Ref		18.5	Ref	
Female	48.7	−2.43	(–5.94, 1.08)	19.6	0.93	(–1.74, 3.60)
Race/Ethnicity						
Hispanic	48.9	0.03	(–4.45, 4.51)	18.1	−1.09	(–4.52, 2.34)
Non-Hispanic Black	52.7	5.47	(–0.14, 11.08)	18.9	−0.001	(–4.26, 4.26)
Non-Hispanic other, multiple, or unknown race/ethnicity	49.7	1.29	(–3.18, 5.77)	19.3	−0.14	(–3.67, 3.40)
Non-Hispanic White	49.1	Ref		19.7	Ref	
Medicaid eligibility type						
Low income	52.0	Ref		20.5	Ref	
Disability	44.5	−8.13***	(–11.94, –4.31)	16.2	−4.57**	(–7.31, –1.83)
Other or unknown eligibility type	52.7	4.46	(–6.71, 15.63)	22.6	5.17	(–4.58, 14.92)
Medicaid plan type						
Comprehensive managed care organization	50.8	Ref		19.8	Ref	
Behavioral health organization, prepaid health plan, or other	47.8	−0.56	(–5.74, 4.62)	19.6	1.00	(–3.19, 5.18)
Primary care case management or fee-for-service	42.5	−9.41***	(–14.98, –3.84)	13.9	−6.67***	(–10.25, –3.09)
Presence of mental health conditions during episode						
Depression only	49.3	Ref		18.9	Ref	
Stress/trauma only	65.5	16.01***	(10.08, 21.95)	34.2	14.99***	(9.53, 20.46)
Anxiety only	33.7	−15.56***	(–19.92, –11.20)	10.0	−9.25***	(–12.24, –6.27)
Two diagnoses	56.6	7.71***	(3.52, 11.89)	21.8	2.76	(–0.58, 6.09)
Three or more diagnoses	69.8	20.52***	(12.52, 28.52)	29.5	10.65**	(2.92, 18.37)
Episode initiation year						
2016	45.4	Ref		18.4	Ref	
2017	47.9	1.75	(–3.62, 7.12)	17.2	−1.78	(–5.97, 2.40)
2018	47.7	2.03	(–3.14, 7.20)	17.9	−0.81	(–4.85, 3.23)
2019	53.5	7.72**	(2.54, 12.91)	20.8	2.19	(–1.92, 6.29)
2020	51.4	4.25	(–0.84, 9.33)	20.6	1.26	(–2.73, 5.26)
Cancer type (first cancer diagnosis)						
Hematological	48.4	Ref		19.2	Ref	
Bone or connective tissue	50.2	1.22	(–3.84, 6.29)	18.4	−1.11	(–4.75, 2.53)
Central nervous system	51.7	2.88	(–1.34, 7.10)	20.8	1.45	(–1.88, 4.78)
Gonadal	50.2	0.41	(–5.24, 6.05)	17.1	−2.38	(–6.60, 1.83)
Time from end-of-cancer-therapy to episode initiation						
90 days to <2 years	49.7	Ref		21.2	Ref	
2-5 years	52.2	2.60	(–3.12, 8.33)	19.4	−1.58	(–6.22, 3.07)
>5 years	47.8	1.01	(–4.78, 6.79)	18.7	−1.43	(–6.10, 3.25)
County-level SDI, Quartiles						
Q1: least deprived areas	53.2	Ref		21.2	Ref	
Q2	50.3	−4.78	(–10.81, 1.25)	20.3	−1.50	(–6.18, 3.17)
Q3	50.4	−4.59	(–10.33, 1.16)	19.1	−2.45	(–6.84, 1.95)
Q4: most deprived areas	47.5	−6.73*	(–12.40, –1.05)	18.0	−2.63	(–7.06, 1.80)
County-level metro status						
Non-metro (including rural) areas	46.7	Ref		18.6	Ref	
Metropolitan areas	50.3	4.09	(–0.84, 9.01)	19.3	1.95	(–1.57, 5.46)
County-level mental health professional shortage areas						
None	45.1	Ref		19.7	Ref	
Partial	50.1	4.89	(–2.48, 12.26)	18.9	−0.26	(–6.31, 5.80)
Full	49.3	7.86	(–0.13, 15.84)	19.9	2.46	(–4.12, 9.04)

Abbreviations: ME = marginal effects; CI = confidence interval; Ref = reference; SDI = Social Deprivation Index.

*n* = 3871 episodes. All covariates listed in this table were included as control variables in the regression models. Regression models also adjusted for state indicators.

a Unadjusted percentage refers to the proportion of individuals within the subgroup specified in each row (eg, females)—that is, using that subgroup as the denominator—who experienced the study outcome (eg, had any mental health visit).

b MEs were interpreted as the model-adjusted difference in the percentage of an outcome (eg, having any mental health visit) between the group of interest (eg, Hispanic survivors) and the reference group (eg, non-Hispanic White survivors) for a given covariate (eg, race/ethnicity), holding all other covariates at their observed values.

* *P *< .05.

** *P *< .01.

*** *P *< .001.

Among children, Hispanic enrollees were less likely than non-Hispanic White peers to have any mental health visit in both unadjusted (60.8% vs 72.8%; Figure S2) and adjusted comparisons (ME = –6.80 ppt, 95% CI = –10.76 to –2.84, *P *< .001; relative reduction = 9.6%; Table 2). This difference was nonsignificant among YAs (Table 3). Additionally, those enrolled due to disability were less likely to have any visit compared with those enrolled due to low income among both children (ME = –7.82 ppt, 95% CI = –11.15 to –4.49, *P *< .001; Table 2) and YAs (ME = –8.13 ppt, 95% CI = –11.94 to –4.31, *P *< .001; Table 3).

When examining county-level covariates among children (Table 2, Figure S2), those living in more deprived areas had lower likelihood of any mental health visit (64.4% in SDI Q4 vs 78.1% for SDI Q1); adjusted model showed a 7.95 ppt reduction (95% CI = –13.04 to –2.87, *P *= .002; relative reduction = 10.7%) in the receipt of any visit for those residing in SDI Q4 compared with SDI Q1 (model-adjusted percentage = 74.29%). Similar differences were seen among YAs in unadjusted (47.5% in SDI Q4 vs 53.2% in SDI Q1) and adjusted comparisons (SDI Q4 vs Q1: ME = –6.73 ppt, 95% CI = –12.40 to –1.05, *P *= .02; relative reduction = 12.4%; Table 3, Figure S2).

### Receipt of ≥4 mental health visits

The likelihood of receiving ≥4 mental health visits during treatment episode was highest among survivors aged 3-11 years (39.8%), followed by those aged 12-17 years (33.2%), 18-26 years (20.2%), and 27-39 years (16.4%; Tables 2-3; Figure S2). Adjusted models with both children and YAs (Table S4) showed a reduction of 4.42 ppt (95% CI = –7.78 to –1.06, *P *= .01; relative reduction = 12.0%), 15.33 ppt (95% CI = –18.82 to –11.83, *P *< .001; relative reduction = 41.6%), and 19.45 ppt (95% CI = –23.65 to –15.26, *P *< .001; relative reduction = 52.8%) in the likelihood of having ≥4 mental health visits for the 12-17 year, 18-26 year, and 27-39 year age groups, respectively, compared with the 3-11 year age group (model-adjusted percentage = 36.83%).

Among children, Hispanic enrollees were less likely to receive ≥4 mental health visits compared with non-Hispanic White peers in unadjusted (27.1% vs 39.6%; Figure S2) and adjusted analyses (ME = –8.73 ppt, 95% CI = –12.78 to –4.68, *P *< .001; relative reduction = 23.0%; Table 2). This difference was statistically nonsignificant among YAs (Table 3). Moreover, those enrolled due to disability were less likely to receive ≥4 visits compared with those enrolled due to low income in both children (ME = –6.16 ppt, 95% CI = –9.47 to –2.86, *P *< .001; Table 2) and YAs (ME = –4.57 ppt, 95% CI = –7.31 to –1.83, *P *= .001; Table 3).

Children living in the most (versus least) deprived counties had a lower likelihood of receiving ≥4 mental health visits (29.7% for SDI Q4 vs 44.6% for SDI Q1; Table 2, Figure S2). Adjusted model among children showed a reduction of 8.80 ppt (95% CI = –14.07 to –3.53; *P *= .001; relative reduction = 21.8%) in the likelihood of receiving ≥4 visits among those living in SDI Q4 compared with SDI Q1 (model-adjusted percentage = 40.35%). This difference was statistically nonsignificant, despite being in a consistent direction, among YAs (Table 3).

### Sensitivity analysis

Our sensitivity analysis excluding states with “high concern” quality of TAF race/ethnicity, enrollment, and plan type variables^44^ contained 2517 treatment episodes from children and 2190 episodes from YAs (Table S5). Findings remained consistent with our main analysis in direction, statistical significance, and magnitude (Tables S6 and S7).

## Discussion

In this multistate study of publicly insured survivors of childhood cancer, we identified notable age-related differences in mental health service utilization after an incident mental health diagnosis, with YA survivors being less likely than child survivors to have any mental health visit or to receive 4 or more visits. Among children aged 3-17 years, Hispanic survivors were less likely than non-Hispanic White peers to receive any mental health visit or to have ≥4 visits. Additionally, higher levels of community deprivation were associated with decreased mental health service use, particularly in child survivors; those residing in the most (vs least) deprived communities were less likely to receive any service or meet the 4-visit threshold.

In our cohort of childhood cancer survivors, 33.2% of adolescents aged 12-17 years and 39.8% of young children aged 3-11 years had ≥4 mental health service visits after their diagnosis for depression, anxiety, or trauma/stress. These estimates are notably lower than those from an earlier claims-based study of all publicly insured children aged 5-17 years in one state, which found that approximately 61% of children with a mental health diagnosis received ≥4 psychosocial visits within 12 weeks of the diagnosis.^37^ The lower rates observed in our cohort suggest that childhood cancer survivors, despite their heightened mental health needs, may be at an increased risk of not receiving needed mental health services. Such increased risk may be due to the long-term physical effects of cancer treatment, such as chronic pain and fatigue,^57^^,^^58^ as well as cancer-related disability, which can hinder the ability to attend frequent in-person appointments. Specialized clinicians, such as psychologists experienced in treating cancer survivors, are rare and often located within cancer centers that may be geographically distant from survivors’ homes.^59^ The geographic barrier, combined with the physical toll of cancer treatment, can further restrict access. More work is needed to identify the barriers to mental health care access and develop strategies for improving access for young survivors, particularly as telehealth expands to address geographic limitations in mental health care delivery.^60^

Although mental health service utilization was low among child survivors, YA survivors are even less likely to use such care, despite guideline recommendations for all survivors.^4^^,^^10^ The transition from pediatric to adult care often leads to a discontinuation of survivorship care, including psychological support.^61^ As YA survivors take on responsibility for their own care, they may lose the parental advocacy they once relied on, perceive stigma around seeking mental health services, and struggle to navigate the complex health care system.^62^^,^^63^ Many YA survivors prioritize returning to normal life activities, such as establishing careers and relationships, which can reduce the perceived need for mental health care.^63^ Health care system factors may also contribute to these age-related disparities. Pediatric cancer centers often transfer survivors during early young adulthood, ending YAs’ access to psychological support in the pediatric oncology setting.^64^ Furthermore, adult care providers may be less attuned to the unique needs of cancer survivors, resulting in fewer referrals to mental health services.^65^

The disparities observed in Hispanic child survivors vs their non-Hispanic White peers are likely multifactorial. The shortage of bilingual providers combined with the language barriers faced by some Hispanic parents may complicate their ability to navigate the mental health system for minor children, restricting patient and family access to an already limited pool of mental health services.^66^^,^^67^ Cultural stigma around mental health,^68^ a lack of culturally competent providers,^66^ and parents’ mistrust of the health-care system^69^ may further deter Hispanic families from seeking mental health care. These barriers, largely at the health care system level, underscore the need for targeted interventions to promote equitable access to psychological support for Hispanic child survivors.

The observed association between area-level deprivation and reduced likelihood of receiving mental health care in children and YAs aligns with prior research showing poorer outcomes for childhood cancer survivors in more deprived communities.^70^ Areas with higher deprivation often have fewer health care facilities and providers available, including those offering mental health services, which likely results in longer travel distances and increased waiting times for appointments.^14^ Additionally, families in deprived areas may face competing psychological stressors such as housing instability and food insecurity, which may take precedence over seeking mental health care.^71^ The stigma surrounding mental health may be more pronounced in these communities, further discouraging service utilization.^68^

### Limitations

Several study limitations should be noted. First, the observational design limits our ability to draw causality. Second, administrative claims data, generated for billing purposes, may introduce measurement errors.^72^ Third, MAX/TAF claims data do not capture out-of-pocket costs for visits, services covered by other payors, or survivors not publicly insured (eg, those privately insured). Fourth, claims data cannot capture undiagnosed mental health needs, an area for future studies. Additionally, our cohort did not include certain childhood cancer types, such as neuroblastoma, that lack validated claims-based algorithms, potentially limiting the generalizability of our findings to other childhood cancers. Lastly, the quality of the TAF data varies by state and variables.^44^ To address this limitation, we narrowed our main analysis to states without high concerns for the TAF Other Service Files and conducted a sensitivity analysis that further restricted the sample to states without high concerns for study variables; all analyses generated consistent results.

## Conclusion

This study provides the first multistate assessment of the realized utilization of mental health services among publicly insured survivors of childhood cancer with an incident mental health diagnosis. We identified low levels of mental health care utilization in these survivors, with significant disparities by age group, ethnicity, and geographic location. These findings highlight the importance of multilevel interventions that can improve access to mental health services for childhood cancer survivors. These can include integrating telehealth services to overcome geographic barriers, increasing funding for mental health resources in deprived areas, and developing training programs for clinicians outside of major pediatric cancer centers to better address the unique needs of these survivors. Future research should investigate unmet mental health care needs and explore the impact of mental health services on quality of life and health outcomes in this young, underserved population.

## Supplementary Material

pkaf099_Supplementary_Data

## Data Availability

The insurance claims data used in this study cannot be shared publicly per the Data Use Agreement with the Centers for Medicare and Medicaid Services.
