# Supplementary material for: Mental health service utilization in publicly insured survivors of childhood cancer: a claims-based analysis
Source: JNCI Cancer Spectr. 2025 Oct 14;9(6):pkaf099. doi: 10.1093/jncics/pkaf099 (PMC12629538; doi:10.1093/jncics/pkaf099)
Supplement: pkaf099_Supplementary_Data [file pkaf099_supplementary_data.pdf]

Supplementary Material

Table S1. Agency for Healthcare Research and Quality Clinical Classification Software (CCS) categories for cancer diagnoses

Table S2. International Classification of Diseases (ICD) diagnosis codes used to define mental health conditions

Table S3. Procedure codes for mental health services

Figure S1. Sample Derivation Flowchart

Figure S2. Unadjusted percentages of any mental health visit and ≥4 mental health visits by key sociodemographic factors

Table S4. Factors associated with utilization of mental health services among all cancer survivors (children and young adults combined)

Table S5. Sensitivity analysis: sample characteristics of mental health treatment episodes in a subset of states with good quality on other services file, race and ethnicity, Medicaid enrollment, and plan type information

Table S6. Sensitivity analysis: factors associated with utilization of mental health services among child survivors ages 3-17 years in a subset of states with good quality on other services file, race and ethnicity, Medicaid enrollment, and plan type information

Table S7. Sensitivity analysis: factors associated with utilization of mental health services among young adult survivors ages 18-39 years in a subset of states with good quality on other services file, race and ethnicity, Medicaid enrollment, and plan type information

Table S1. Agency for Healthcare Research and Quality Clinical Classification Software (CCS) categories for cancer diagnoses

CCS Categories	Cancer Types
37, 38, 39	Hematologic cancers (leukemia, lymphoma)
35	Central nervous system
21	Bone or connective tissue
27, 30	Gonadal Cancer

Notes: Detailed ICD codes for each CCS category can be downloaded from:
https://www.hcup-us.ahrq.gov/toolssoftware/ccs/ccs.jsp
https://www.hcup-us.ahrq.gov/toolssoftware/ccs10/ccs10.jsp

Table S2. International Classification of Diseases (ICD) diagnosis codes used to define mental health conditions

A. ICD diagnosis codes for the three specific mental health conditions of interest

Mental Health Conditions of Interest	ICD-10 Diagnosis Codes
Anxiety	F930, F940, F40210, F40218, F40220, F40228, F40230, F40231, F40232, F40233, F40240, F40241, F40242, F40243, F40248, F40290, F40291, F40298, F4010, F410, F4000, F411, F064, F418, F419
Depressive disorder	F3481, F320, F321, F322, F323, F324, F325, F329, F330, F331, F332, F333, F3340, F3341, F3342, F339, F341, F3281, F0631, F0630, F0632, F0633, F0634, F3289, F32A, F39
Trauma/stress	F941, F942, F4310, F430, F4320, F4321, F4322, F4323, F4324, F4325, F4329, F438, F438, F439

B. ICD diagnosis codes for all mental health conditions used to define the "exclusion period"

Mental Health Conditions	ICD-10 Diagnosis Codes
Attention-Deficit/Hyperactivity Disorder (ADHD)	F900, F901, F902, F908, F909
Anxiety	F4000, F4001, F4002, F4010, F4011, F40210, F40218, F40220, F40228, F40230, F40231, F40232, F40233, F40240, F40241, F40242, F40243, F40248, F40290, F40291, F40298, F408, F409, F410, F411, F413, F418, F419, F064, F930, F940
Autism	F845, F840
Depressive Disorder	F0631, F0632, F320, F321, F322, F323, F324, F325, F3281, F329, F330, F331, F332, F333, F3340, F3341, F3342, F339, F341, F3481, F3289, F338, F328, F602, F631, F632, F6381, F6389, F639, F910, F911, F912, F918, F919, F913
Disruptive Disorder	F602, F631, F632, F6381, F6389, F639, F910, F911, F912, F918, F919, F913
Obsessive Disorder	F633, F42, F422, F423, F428, F429, F424
Trauma/stress	F430, F4310, F4311, F4312, F4320, F4321, F4322, F4323, F4324, F4325, F4329, F438, F439, F941, F942, R457
Eating	3071, 30750, 30751, 30752, 30753, 30754, 30759
Schizophrenia	F060, F062, F200, F201, F202, F203, F205, F2081, F2089, F209, F21, F22, F23, F24, F250, F251, F258, F259, F28, F29, F601
Bipolar	F304, F308, F309, F310, F3110, F3111, F3112, F3113, F312, F3130, F3131, F3132, F314, F315, F3160, F3161, F3162, F3163, F3164, F3170, F3171, F3172, F3173, F3174, F3175, F3176, F3177, F3178, F3181, F3189, F319, F340
Personality	F600, F604, F605, F606, F607, F6081, F6089, F609, F69, F603
Other neuro-developmental	F70, F71, F72, F73, F78, F79, F800, F801, F802, F804, F8081, F8082, F8089, F809, F810, F812, F8181, F8189, F819, F82, F843, F848, F849, F842, F88, F89, F950, F951, F952, F958, F959, F984, F985, R480, R4183
Other	F0630, F348, F3489, F349, F39, F488, F489, F642, F938, F939, F948, F949, F980, F981, F988, F989

Notes: All the codes in this table were used to ensure no mental health diagnosis during the 90-day exclusion period.

Table S3. Procedure codes for mental health services

Category	Procedure Codes
Applied behavior analysis	0359T, 0360T, 0361T, 0362T, 0363T, 0364T, 0365T, 0366T, 0367T, 0368T, 0369T, 0370T, 0371T, 0372T, 0373T, 0374T, 97153, 97154, 97155, 97156, 97157, 97158
Psychosocial intervention	90804, 90805, 90806, 90807, 90808, 90809, 90810, 90811, 90812, 90813, 90814, 90815, 90833, 90838, 90862, 90863, 90832, 90834, 90836, 90837, 90839, 90840, 90845, 90846, 90847, 90849, 90853, 90857, 90875, 90876, 96153, 96164, 96165, 96152, 96154, 96155, 96158, 96159, 96167, 96168, 96170, 96171, 99354, 99355, 99510, G0410, G0411, H0004, H2012, H2033
Medication management	90792, M0064
Mental health assessment	90791, 90801, 90802, 96127, 96150, 96151, 96156, 96160, 96161, 97151, 97152, G0444, H0031
Neuropsychological & psychological testing	96100, 96101, 96102, 96103, 96116, 96117, 96118, 96119, 96120, 96121, 96125, 96130, 96131, 96132, 96133, 96136, 96137, 96138, 96139, 96146
Other mental health interventions	90880, 97533, G0409, G0469, G0470, G0515, H0036, H0037, H0046, H2014, H2015, H2016, H2017, H2018, H2019, H2020, H2021, H2022, H2027, H2030, H2031, H2032, S9480, S9484, S9485, T1027

Figure S1. Sample Derivation Flowchart

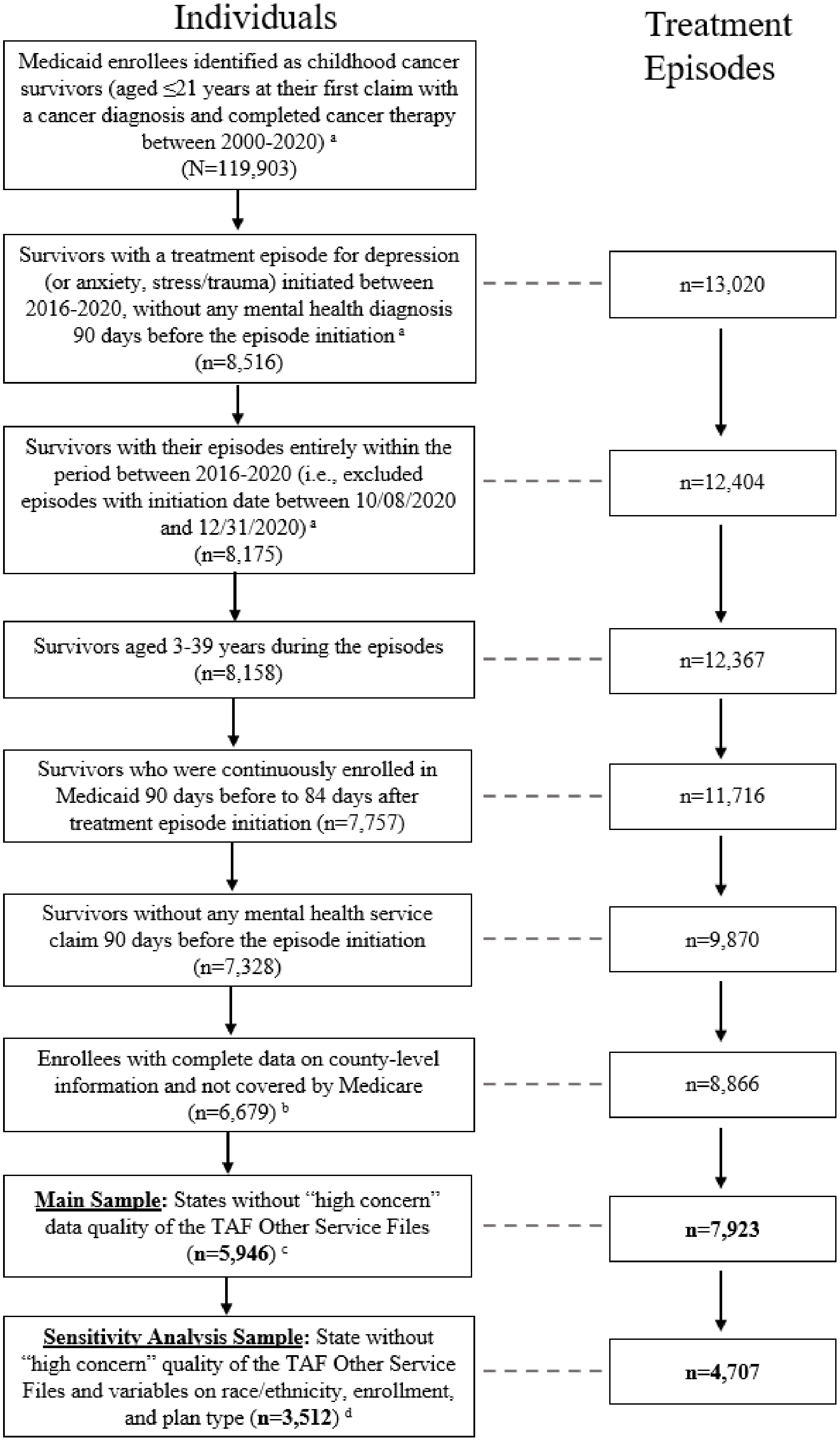

Notes: [a] Our study cohort consists of survivors of childhood cancer with mental health treatment episodes. Childhood cancer survivors were identified using MAX and TAF data in 2000-2020 to ensure comprehensive capture of cancer diagnoses, cancer therapies, and thus, all eligible survivors diagnosed with and treated for childhood cancer. In contrast, mental health diagnoses and subsequent mental health treatment episodes in the survivorship phase (the period after completion of cancer therapy) were defined using TAF data from 2016-2020. This approach (1) ensured variation across individuals in the interval from cancer therapy completion to their mental health treatment episode initiation—allowing comparison of early versus long-term survivors—and (2) avoided potential inconsistencies in key measures such as Medicaid eligibility and plan type between the MAX and TAF data formats, given that all states had fully transitioned to TAF data by 2016.

[b] We excluded 337 individuals missing county identifiers and/or missing county-level variables. We also excluded 312 individuals who were dual enrolled in Medicare.

[c] The main sample included 43 states (AL, AK, AZ, AR, CA, CO, CT, DE, GA, HI, ID, IL, IN, IA, KS, KY, LA, ME, MD, MI, MO, MT, NE, NV, NH, NM, NY, NC, ND, OH, OK, OR, PA, SC, SD, TN, TX, VT, VA, WA, WV, WI, WY) and DC that did not have "high concern" data quality issues in the TAF Other Services File.

[d] The sensitivity analysis sample included 17 states (AK, CA, DE, FL, GA, IL, IN, KY, ME, MN, NH, NM, NC, OH, TX, VI, WI) that did not have "high concern" data quality issues in the TAF Other Services File and variables on race/ethnicity, enrollment, and plan type.

Figure S2. Unadjusted percentages of any mental health visit and ≥4 mental health visits by key sociodemographic factors

A. Unadjusted percentages of any mental health visit and ≥4 mental health visits by age group

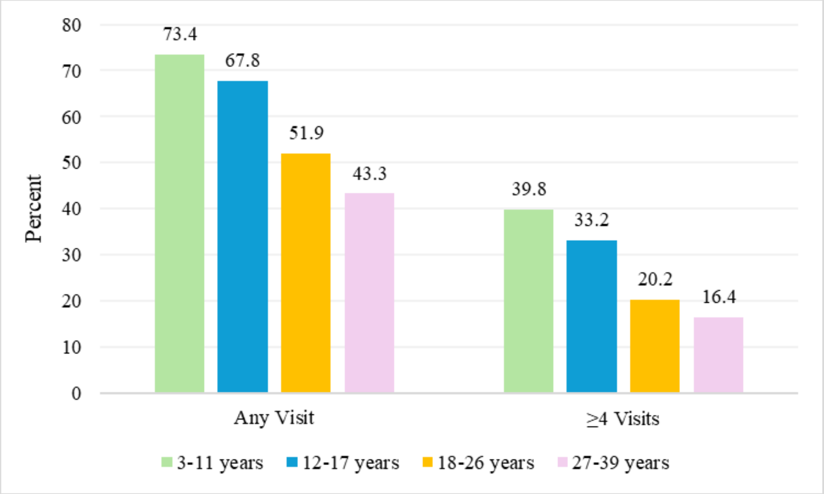

B. Unadjusted percentages of any mental health visit and ≥4 mental health visits by race/ethnicity among children ages 3-17

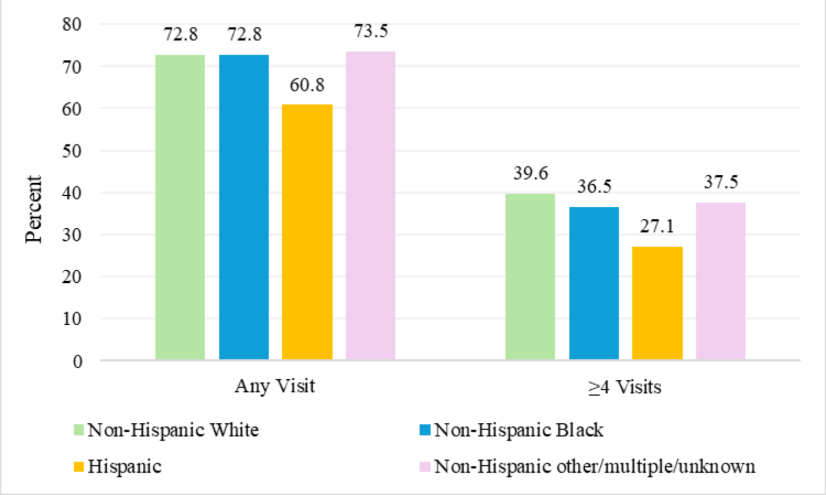

C. Unadjusted percentages of any mental health visit and ≥4 mental health visits by race/ethnicity among young adults ages 18-39

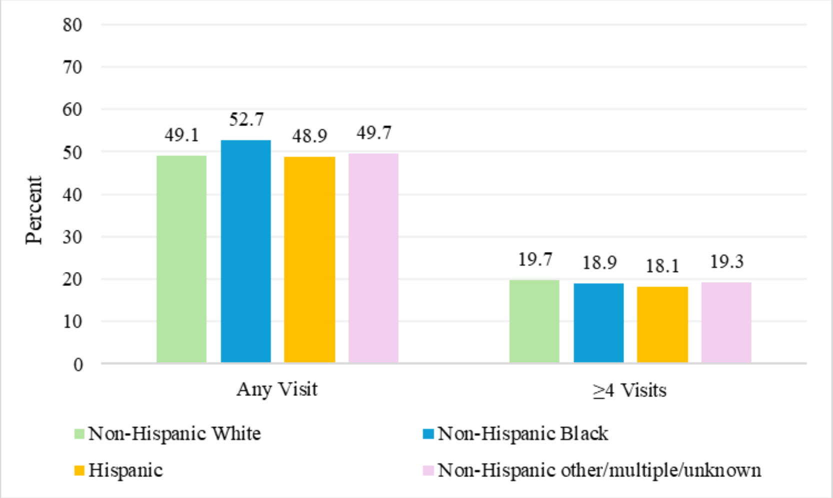

D. Unadjusted percentages of any mental health visit and ≥4 mental health visits by Social Deprivation Index (SDI) quartiles among children ages 3-17

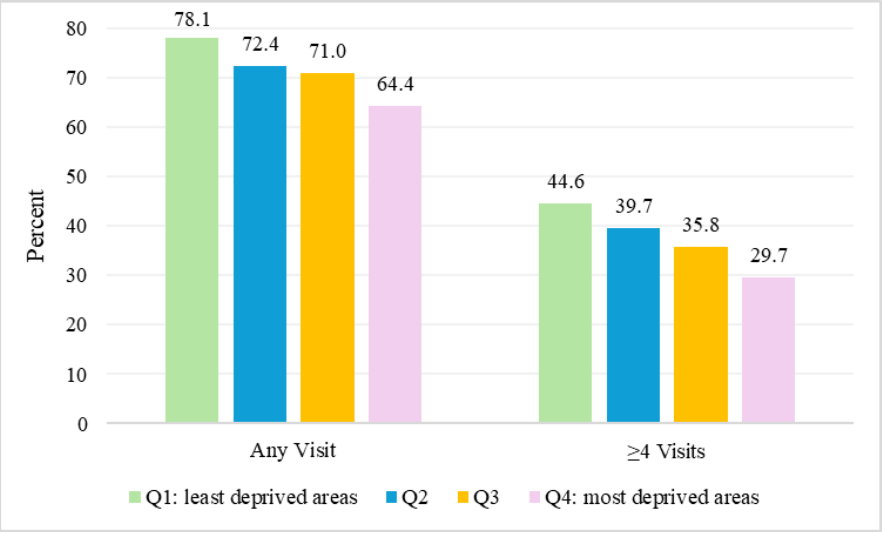

E. Unadjusted percentages of any mental health visit and ≥4 mental health visits by SDI quartiles among young adults ages 18-39

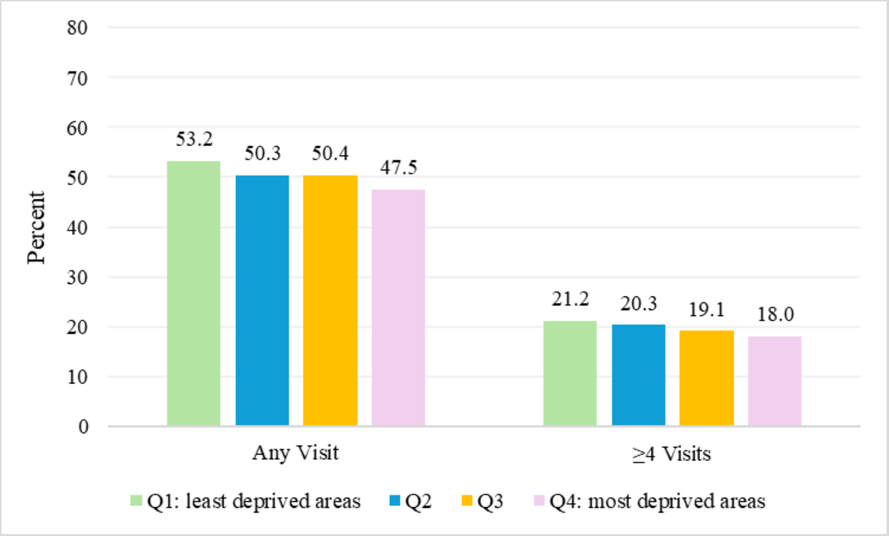

Table S4. Factors associated with utilization of mental health services among all cancer survivors (children and young adults combined)

Characteristics	Any Mental Health Visit				≥4 Mental Health Visits			
	Unadjusted Percentage (%)[1]	Adjusted Differences			Unadjusted Percentage (%)[1]	Adjusted Differences		
		ME[2]	95% CI	*P*		ME[2]	95% CI	*P*
Age group at index date								
3-11 years	73.4	Ref			39.8	Ref		
12-17 years	67.8	-4.32	(-7.60, -1.03)	0.010	33.2	-4.42	(-7.78, -1.06)	0.010
18-26 years	51.9	-17.23	(-20.73, -13.73)	<0.001	20.2	-15.33	(-18.82, -11.83)	<0.001
27-39 years	43.3	-25.93	(-30.67, -21.19)	<0.001	16.4	-19.45	(-23.65, -15.26)	<0.001
Sex								
Male	61.1	Ref			28.1	Ref		
Female	59.0	-1.10	(-3.40, 1.20)	0.349	27.1	0.19	(-1.86, 2.24)	0.854
Race/Ethnicity								
Hispanic	55.7	-4.11	(-7.10, -1.11)	0.007	23.2	-5.34	(-8.00, -2.68)	<0.001
Non-Hispanic Black	61.1	4.53	(0.57, 8.49)	0.025	26.3	-0.05	(-3.76, 3.65)	0.977
Non-Hispanic other, multiple, or unknown race/ethnicity	62.8	1.83	(-1.11, 4.76)	0.223	29.3	-0.35	(-3.05, 2.35)	0.800
Non-Hispanic White	60.5	Ref			29.3	Ref		
Medicaid eligibility type								
Low income	62.5	Ref			29.4	Ref		
Disability	53.9	-8.15	(-10.70, -5.61)	<0.001	23.2	-5.30	(-7.46, -3.14)	<0.001
Other or unknown eligibility type	65.7	6.63	(-0.30, 13.56)	0.061	30.4	4.56	(-2.76, 11.88)	0.223
Medicaid plan type								
Comprehensive managed care organization	60.4	Ref			28.0	Ref		
Behavioral health organization, prepaid health plan, or other	62.9	2.87	(-0.40, 6.13)	0.085	28.9	0.48	(-2.64, 3.60)	0.762
Primary care case management or fee-for-service	52.5	-10.52	(-14.33, -6.70)	<0.001	21.8	-7.72	(-10.66, -4.78)	<0.001
Presence of mental health conditions during episode								
Depression only	54.7	Ref			22.9	Ref		
Stress/trauma only	77.4	16.14	(12.79, 19.49)	<0.001	42.2	12.25	(9.12, 15.37)	<0.001
Anxiety only	43.3	-12.38	(-15.65, -9.10)	<0.001	17.6	-6.52	(-9.15, -3.89)	<0.001
Two diagnoses	63.4	9.37	(6.31, 12.43)	<0.001	27.2	4.67	(1.87, 7.46)	0.001
Three or more diagnoses	77.8	22.14	(16.69, 27.60)	<0.001	40.2	16.77	(10.45, 23.09)	<0.001
Episode initiation year								
2016	58.0	Ref			25.9	Ref		
2017	58.1	-0.13	(-3.70, 3.44)	0.943	24.9	-0.96	(-4.19, 2.27)	0.562
2018	58.7	0.86	(-2.57, 4.28)	0.624	26.8	0.83	(-2.29, 3.95)	0.602
2019	63.6	5.33	(1.93, 8.73)	0.002	30.4	4.12	(1.01, 7.24)	0.010
2020	59.6	1.90	(-1.55, 5.35)	0.280	28.1	2.66	(-0.49, 5.81)	0.097

Cancer type (first cancer diagnosis)								
Hematologic	59.7	Ref			27.6	Ref		
Bone or connective tissue	60.0	-0.32	(-3.69, 3.05)	0.854	26.6	-1.11	(-3.98, 1.76)	0.448
Central nervous system	62.3	2.42	(-0.32, 5.16)	0.084	29.9	2.05	(-0.45, 4.55)	0.108
Gonadal	52.5	-1.36	(-5.95, 3.23)	0.561	19.9	-3.02	(-7.16, 1.12)	0.153
Time from end-of-cancer-therapy to episode initiation								
90 days to <2 years	61.3	Ref			29.6	Ref		
2-5 years	63.1	3.32	(0.04, 6.60)	0.047	29.6	1.08	(-1.85, 4.01)	0.471
>5 years	56.0	2.63	(-0.89, 6.16)	0.143	24.6	1.05	(-2.15, 4.25)	0.520
County-level SDI, Quartiles								
Q1: least deprived areas	66.1	Ref			33.3	Ref		
Q2	61.8	-3.85	(-7.88, 0.18)	0.061	30.4	-1.62	(-5.27, 2.02)	0.382
Q3	60.6	-3.90	(-7.71, -0.08)	0.045	27.4	-3.48	(-6.96, -0.01)	0.050
Q4: most deprived areas	56.2	-7.42	(-11.24, -3.60)	<0.001	24.0	-5.95	(-9.41, -2.50)	<0.001
County-level metro status								
Non-metro (including) rural areas	59.6	Ref			27.2	Ref		
Metropolitan areas	59.9	3.73	(0.40, 7.05)	0.028	27.6	2.67	(-0.10, 5.45)	0.059
County-level mental health professional shortage areas								
None	63.6	Ref			33.3	Ref		
Partial	58.6	-2.23	(-7.18, 2.73)	0.378	26.6	-3.45	(-8.14, 1.24)	0.149
Full	62.5	4.14	(-1.15, 9.44)	0.125	28.7	0.10	(-4.94, 5.15)	0.969

Abbreviation: ME = Marginal Effects. CI = confidence interval. Ref = reference. SDI = Social Deprivation Index.
N=7923 episodes. All covariates listed in this table were included as control variables in the regression models. Regression models also adjusted for state indicators.

[1] Unadjusted percentage refers to the proportion of individuals within the subgroup specified in each row (e.g., females)—that is, using that subgroup as the denominator—who experienced the study outcome (e.g., had any mental health visit).

[2] MEs were interpreted as the model-adjusted difference in the percentage of an outcome (e.g., having any mental health visit) between the group of interest (e.g., Hispanic survivors) and the reference group (e.g., non-Hispanic White survivors) for a given covariate (e.g., race/ethnicity), holding all other covariates at their observed values.

Table S5. Sensitivity analysis: sample characteristics of mental health treatment episodes in a subset of states with good quality on other services file, race and ethnicity, Medicaid enrollment, and plan type information

Characteristics	Children ages 3-17 years		Young adults ages 18-39 years	
	N	%	N	%
Total observations	2517		2190	
Age groups at index date				
3-11 years	846	33.6	.	.
12-17 years	1671	66.4	.	.
18-26 years	.	.	1611	73.6
27-39 years	.	.	579	26.4
Sex				
Male	1120	44.5	892	40.7
Female	1397	55.5	1298	59.3
Race/Ethnicity				
Hispanic	933	37.1	634	28.9
Non-Hispanic Black	224	8.9	284	13.0
Non-Hispanic other, multiple, or unknown race/ethnicity	378	15.0	279	12.7
Non-Hispanic White	982	39.0	993	45.3
Medicaid eligibility type				
Low income	1666	66.2	1342	61.3
Disability	818	32.5	815	37.2
Other or unknown eligibility type	33	1.3	33	1.5
Medicaid plan type				
Comprehensive managed care organization	2019	80.2	1724	78.7
Behavioral health organization, prepaid health plan, or other plan types	250	9.9	221	10.1
Primary care case management or fee-for-service	248	9.9	245	11.2
Presence of mental health conditions during episode				
Depression only	605	24.0	651	29.7
Stress/trauma only	842	33.5	190	8.7
Anxiety only	535	21.3	602	27.5
Two diagnoses	492	19.5	674	30.8
Three or more diagnoses	43	1.7	73	3.3
Episode initiation year				
2016	377	15.0	328	15.0
2017	480	19.1	392	17.9
2018	547	21.7	446	20.4
2019	636	25.3	532	24.3
2020	477	19.0	492	22.5

Cancer type (first cancer diagnosis)				
Hematologic	1392	55.3	1168	53.3
Bone or connective tissue	378	15.0	295	13.5
Central nervous system	652	25.9	490	22.4
Gonadal	95	3.8	237	10.8
Time from end-of-cancer-therapy to episode initiation				
90 days to <2 years	418	16.6	210	9.6
2-5 years	1366	54.3	829	37.9
>5 years	733	29.1	1151	52.6
County-level Social Deprivation Index (SDI), Quartiles				
Q1: least deprived areas	261	10.4	231	10.5
Q2	468	18.6	429	19.6
Q3	634	25.2	544	24.8
Q4: most deprived areas	1154	45.8	986	45.0
County-level metro status				
Non-metro (including rural) areas	402	16.0	388	17.7
Metropolitan areas	2115	84.0	1802	82.3
County-level mental health professional shortage areas				
None	124	4.9	103	4.7
Partial	585	23.2	544	24.8
Full	1808	71.8	1543	70.5

Table S6. Sensitivity analysis: factors associated with utilization of mental health services among child survivors ages 3-17 years in a subset of states with good quality on other services file, race and ethnicity, Medicaid enrollment, and plan type information

Characteristics	Any Mental Health Visit				≥4 Mental Health Visits			
	Unadjusted Percentage (%)[1]	Adjusted Differences			Unadjusted Percentage (%)[1]	Adjusted Differences		
		ME[2]	95% CI	*P*		ME[2]	95% CI	*P*
Age groups at index date								
3-11 years	70.3	Ref			34.9	Ref		
12-17 years	66.1	-2.87	(-7.06, 1.32)	0.180	29.0	-4.91	(-9.17, -0.65)	0.024
Sex								
Male	66.9	Ref			28.8	Ref		
Female	68.1	1.43	(-2.36, 5.22)	0.459	32.7	4.45	(0.72, 8.18)	0.019
Race/Ethnicity								
Hispanic	58.6	-9.33	(-14.11, -4.55)	<0.001	23.2	-10.61	(-15.45, -5.76)	<0.001
Non-Hispanic Black	75.0	3.57	(-3.83, 10.97)	0.344	33.9	-1.97	(-9.46, 5.51)	0.605
Non-Hispanic other, multiple, or unknown race/ethnicity	69.6	-0.35	(-6.02, 5.33)	0.904	27.8	-7.65	(-13.44, -1.86)	0.010
Non-Hispanic White	73.5	Ref			39.0	Ref		
Medicaid eligibility type								
Low-income	70.5	Ref			33.1	Ref		
Disability	61.6	-7.82	(-11.95, -3.69)	<0.001	26.8	-4.82	(-8.85, -0.79)	0.019
Other or unknown eligibility type	66.7	-0.72	(-15.19, 13.75)	0.922	27.3	-2.98	(-18.96, 13.00)	0.714
Medicaid plan type								
Comprehensive managed care organization	67.4	Ref			30.4	Ref		
Behavioral health organization, prepaid health plan, or other	78.4	9.13	(3.30, 14.96)	0.002	39.6	7.83	(1.29, 14.37)	0.019
Primary care case management or fee-for-service	58.1	-14.44	(-21.09, -7.79)	<0.001	27.4	-5.84	(-11.41, -0.27)	0.040
Presence of mental health conditions during episode								
Depression only	58.0	Ref			22.6	Ref		
Stress/trauma only	79.1	18.69	(13.74, 23.65)	<0.001	39.6	14.33	(9.38, 19.29)	<0.001
Anxiety only	52.7	-8.19	(-14.29, -2.08)	0.009	23.9	-1.41	(-6.68, 3.85)	0.598
Two diagnoses	73.6	13.77	(8.12, 19.41)	<0.001	31.9	7.46	(2.04, 12.88)	0.007
Three or more diagnoses	90.7	28.25	(17.23, 39.27)	<0.001	58.1	29.93	(14.48, 45.38)	<0.001
Episode initiation year								
2016	67.1	Ref			26.8	Ref		
2017	65.8	-2.34	(-8.33, 3.65)	0.444	27.7	0.34	(-5.37, 6.05)	0.907
2018	66.2	-0.43	(-6.24, 5.37)	0.883	31.8	5.19	(-0.59, 10.98)	0.078
2019	69.0	1.93	(-3.70, 7.57)	0.502	33.5	6.64	(0.92, 12.37)	0.023
2020	69.2	0.52	(-5.61, 6.64)	0.869	33.3	5.71	(-0.21, 11.63)	0.059

Cancer type (first cancer diagnosis)								
Hematologic	68.3	Ref			31.3	Ref		
Bone or connective tissue	68.8	-2.36	(-7.76, 3.04)	0.393	28.3	-3.97	(-9.08, 1.15)	0.129
Central nervous system	66.4	-2.27	(-6.66, 2.13)	0.313	33.1	1.79	(-2.67, 6.25)	0.432
Gonadal	58.9	-8.82	(-18.97, 1.33)	0.088	23.2	-6.40	(-15.83, 3.03)	0.183
Time from end-of-cancer-therapy to episode initiation								
90 days to <2 years	64.1	Ref			29.0	Ref		
2-5 years	68.4	4.74	(-0.21, 9.70)	0.061	31.3	2.45	(-2.42, 7.33)	0.324
>5 years	67.8	4.57	(-1.02, 10.17)	0.109	31.7	4.14	(-1.55, 9.83)	0.154
County-level SDI, Quartiles								
Q1: least deprived areas	80.1	Ref			44.8	Ref		
Q2	69.4	-7.70	(-15.09, -0.31)	0.041	35.0	-5.59	(-13.13, 1.95)	0.146
Q3	67.5	-7.30	(-14.12, -0.48)	0.036	30.8	-6.93	(-14.13, 0.27)	0.059
Q4: most deprived areas	64.0	-9.42	(-16.06, -2.79)	0.005	26.3	-10.10	(-17.19, -3.01)	0.005
County-level metro status								
Non-metro (including rural) areas	76.9	Ref			38.6	Ref		
Metropolitan areas	65.8	-0.63	(-6.45, 5.19)	0.832	29.6	0.63	(-4.93, 6.18)	0.825
County-level mental health professional shortage areas								
None	83.1	Ref			45.2	Ref		
Partial	63.6	-12.04	(-20.30, -3.78)	0.004	28.0	-8.44	(-17.40, 0.52)	0.065
Full	76.6	-0.64	(-9.42, 8.14)	0.886	37.3	-1.05	(-10.65, 8.56)	0.831

Abbreviation: ME = Marginal Effects. CI = confidence interval. Ref = reference. SDI = Social Deprivation Index.
N=2517 episodes. All covariates listed in this table were included as control variables in the regression models. Regression models also adjusted for state indicators.

[1] Unadjusted percentage refers to the proportion of individuals within the subgroup specified in each row (e.g., females)—that is, using that subgroup as the denominator—who experienced the study outcome (e.g., had any mental health visit).

[2] MEs were interpreted as the model-adjusted difference in the percentage of an outcome (e.g., having any mental health visit) between the group of interest (e.g., Hispanic survivors) and the reference group (e.g., non-Hispanic White survivors) for a given covariate (e.g., race/ethnicity), holding all other covariates at their observed values.

Table S7. Sensitivity analysis: factors associated with utilization of mental health services among young adult survivors ages 18-39 years in a subset of states with good quality on other services file, race and ethnicity, Medicaid enrollment, and plan type information

Characteristics	Any Mental Health Visit				≥4 Mental Health Visits			
	Unadjusted Percentage (%)[1]	Adjusted Differences			Unadjusted Percentage (%)[1]	Adjusted Differences		
		ME[2]	95% CI	*P*		ME[2]	95% CI	*P*
Age groups at index date								
18-26 years	50.4	Ref			18.1	Ref		
27-39 years	43.2	-7.55	(-12.98, -2.12)	0.006	17.6	-1.04	(-5.07, 2.98)	0.612
Sex								
Male	48.9	Ref			16.9	Ref		
Female	48.2	-1.43	(-5.96, 3.10)	0.536	18.7	1.40	(-1.96, 4.76)	0.414
Race/Ethnicity								
Hispanic	46.7	-1.44	(-7.10, 4.22)	0.619	15.9	-3.51	(-7.71, 0.68)	0.101
Non-Hispanic Black	49.6	4.52	(-2.51, 11.55)	0.207	13.7	-4.80	(-9.94, 0.34)	0.067
Non-Hispanic other, multiple, or unknown race/ethnicity	49.8	4.14	(-2.89, 11.18)	0.249	19.0	0.91	(-4.85, 6.67)	0.757
Non-Hispanic White	48.9	Ref			20.2	Ref		
Medicaid eligibility type								
Low-income	50.9	Ref			19.6	Ref		
Disability	43.7	-8.49	(-13.43, -3.55)	0.001	14.9	-4.59	(-8.13, -1.06)	0.011
Other or unknown eligibility type	69.7	20.72	(6.53, 34.92)	0.004	30.3	13.87	(-3.04, 30.77)	0.108
Medicaid plan type								
Comprehensive managed care organization	50.5	Ref			19.0	Ref		
Behavioral health organization, prepaid health plan, or other	43.0	-5.39	(-13.40, 2.61)	0.187	14.5	-4.02	(-9.69, 1.64)	0.164
Primary care case management or fee-for-service	39.6	-14.24	(-20.69, -7.79)	<0.001	14.3	-6.02	(-10.48, -1.55)	0.008
Presence of mental health conditions during episode								
Depression only	48.2	Ref			17.1	Ref		
Stress/trauma only	60.5	12.82	(4.72, 20.91)	0.002	30.0	12.64	(5.38, 19.91)	0.001
Anxiety only	33.6	-15.15	(-20.81, -9.49)	<0.001	10.1	-7.97	(-11.85, -4.10)	<0.001
Two diagnoses	56.4	8.48	(2.93, 14.03)	0.003	21.1	3.07	(-1.21, 7.36)	0.159
Three or more diagnoses	69.9	21.07	(10.26, 31.87)	<0.001	31.5	13.87	(3.56, 24.18)	0.008
Episode initiation year								
2016	47.3	Ref			15.9	Ref		
2017	45.9	-2.60	(-9.80, 4.60)	0.479	15.3	-0.98	(-6.20, 4.24)	0.713
2018	45.5	-2.81	(-9.88, 4.26)	0.436	16.6	0.37	(-4.83, 5.57)	0.888
2019	52.4	4.56	(-2.39, 11.51)	0.199	20.7	4.84	(-0.41, 10.09)	0.071
2020	49.8	1.26	(-5.67, 8.19)	0.722	19.9	3.99	(-1.30, 9.28)	0.139

Cancer type (first cancer diagnosis)								
Hematologic	47.4	Ref			18.0	Ref		
Bone or connective tissue	48.5	0.52	(-6.33, 7.37)	0.881	16.6	-1.06	(-5.96, 3.84)	0.672
Central nervous system	50.0	2.01	(-3.60, 7.62)	0.482	18.6	0.90	(-3.40, 5.20)	0.683
Gonadal	50.6	0.96	(-6.54, 8.46)	0.803	18.6	-0.09	(-5.74, 5.57)	0.976
Time from end-of-cancer-therapy to episode initiation								
90 days to <2 years	44.8	Ref			19.5	Ref		
2-5 years	51.5	6.70	(-0.68, 14.08)	0.075	17.6	-1.91	(-7.84, 4.01)	0.527
>5 years	47.0	4.68	(-2.77, 12.13)	0.218	18.0	-1.43	(-7.33, 4.47)	0.635
County-level SDI, Quartiles								
Q1: least deprived areas	56.7	Ref			23.8	Ref		
Q2	50.1	-7.33	(-15.63, 0.97)	0.083	19.6	-2.58	(-9.17, 4.01)	0.443
Q3	47.6	-9.41	(-17.45, -1.37)	0.022	16.7	-4.71	(-10.79, 1.37)	0.129
Q4: most deprived areas	46.3	-9.04	(-16.77, -1.31)	0.022	16.6	-3.27	(-9.43, 2.88)	0.298
County-level metro status								
Non-metro (including rural) areas	49.2	Ref			20.4	Ref		
Metropolitan areas	48.3	0.27	(-6.66, 7.20)	0.940	17.5	-1.49	(-6.73, 3.75)	0.578
County-level mental health professional shortage areas								
None	46.6	Ref			22.3	Ref		
Partial	48.3	1.87	(-8.68, 12.41)	0.729	17.4	-4.44	(-13.71, 4.83)	0.348
Full	49.3	3.77	(-7.61, 15.15)	0.516	18.8	-4.50	(-14.30, 5.31)	0.369

Abbreviation: ME = Marginal Effects. CI = confidence interval. Ref = reference. SDI = Social Deprivation Index.
N=2190 episodes. All covariates listed in this table were included as control variables in the regression models. Regression models also adjusted for state indicators.
[1] Unadjusted percentage refers to the proportion of individuals within the subgroup specified in each row (e.g., females)—that is, using that subgroup as the denominator—who experienced the study outcome (e.g., had any mental health visit).
[2] MEs were interpreted as the model-adjusted difference in the percentage of an outcome (e.g., having any mental health visit) between the group of interest (e.g., Hispanic survivors) and the reference group (e.g., non-Hispanic White survivors) for a given covariate (e.g., race/ethnicity), holding all other covariates at their observed values.
